# Supplementary material for: Smooth Interpolating Curves with Local Control and Monotone Alternating Curvature
Source: Comput Graph Forum. 2022 Oct 6;41(5):25–38. doi: 10.1111/cgf.14600 (PMC9827861; doi:10.1111/cgf.14600)
Supplement: Supplementary file 1 — Supplement Material [file CGF-41-25-s001.zip › Local-Smooth-Interpolating-MonoCurvature/extern/clothoids/submodules/Utils/docs/api-cpp/program_listing_file_Utils_Malloc.hxx.html]

Program Listing for File Malloc.hxx — Utils v1.2

### Navigation

- index
- toc
- Utils »
- Program Listing for File Malloc.hxx

# Program Listing for File Malloc.hxx¶

↰ Return to documentation for file (`Utils/Malloc.hxx`)

```
/*--------------------------------------------------------------------------*\
 |                                                                          |
 |  Copyright (C) 2020                                                      |
 |                                                                          |
 |         , __                 , __                                        |
 |        /|/  \               /|/  \                                       |
 |         | __/ _   ,_         | __/ _   ,_                                |
 |         |   \|/  /  |  |   | |   \|/  /  |  |   |                        |
 |         |(__/|__/   |_/ \_/|/|(__/|__/   |_/ \_/|/                       |
 |                           /|                   /|                        |
 |                           \|                   \|                        |
 |                                                                          |
 |      Enrico Bertolazzi                                                   |
 |      Dipartimento di Ingegneria Industriale                              |
 |      Universita` degli Studi di Trento                                   |
 |      email: enrico.bertolazzi@unitn.it                                   |
 |                                                                          |
\*--------------------------------------------------------------------------*/


#pragma once

#ifndef DOXYGEN_SHOULD_SKIP_THIS
#ifndef UTILS_MALLOC_dot_HXX
#define UTILS_MALLOC_dot_HXX
#endif

/*\
:|:    ____            _       _             __
:|:   / ___| _     _  (_)_ __ | |_ ___ _ __ / _| __ _  ___ ___
:|:  | |   _| |_ _| |_| | '_ \| __/ _ \ '__| |_ / _` |/ __/ _ \
:|:  | |__|_   _|_   _| | | | | ||  __/ |  |  _| (_| | (_|  __/
:|:   \____||_|   |_| |_|_| |_|\__\___|_|  |_|  \__,_|\___\___|
\*/

namespace Utils {

  #ifndef DOXYGEN_SHOULD_SKIP_THIS
  using std::int64_t;
  using std::string;
  using std::mutex;
  #endif


  extern mutex MallocMutex;

  extern int64_t CountAlloc;
  extern int64_t CountFreed;
  extern int64_t AllocatedBytes;
  extern int64_t MaximumAllocatedBytes;
  extern bool    MallocDebug;

  string outBytes( size_t nb );

  /*\
  :|:   __  __       _ _
  :|:  |  \/  | __ _| | | ___   ___
  :|:  | |\/| |/ _` | | |/ _ \ / __|
  :|:  | |  | | (_| | | | (_) | (__
  :|:  |_|  |_|\__,_|_|_|\___/ \___|
  \*/

  template <typename T>
  class Malloc {
  public:
    typedef T valueType;

  private:

    string      m_name;
    size_t      m_numTotValues;
    size_t      m_numTotReserved;
    size_t      m_numAllocated;
    valueType * m_pMalloc;

    Malloc(Malloc<T> const &) = delete; // blocco costruttore di copia
    Malloc<T> const & operator = (Malloc<T> &) const = delete; // blocco copia

    void allocate_internal( size_t n );
    void memory_exausted( size_t sz );

  public:

    explicit
    Malloc( string const & name );

    ~Malloc() { hard_free(); }

    void allocate( size_t n );

    void reallocate( size_t n );

    void free(void) { m_numTotValues = m_numAllocated = 0; }

    void hard_free(void);

    size_t size(void) const { return m_numTotValues; }

    T * operator () ( size_t sz ) {
      size_t offs = m_numAllocated;
      m_numAllocated += sz;
      if ( m_numAllocated > m_numTotValues ) memory_exausted( sz );
      return m_pMalloc + offs;
    }

    T * malloc( size_t n );
    T * realloc( size_t n );

    bool is_empty() const { return m_numAllocated >= m_numTotValues; }

    void must_be_empty( char const * const where ) const;
  };

  extern template class Malloc<char>;
  extern template class Malloc<uint16_t>;
  extern template class Malloc<int16_t>;
  extern template class Malloc<uint32_t>;
  extern template class Malloc<int32_t>;
  extern template class Malloc<uint64_t>;
  extern template class Malloc<int64_t>;
  extern template class Malloc<float>;
  extern template class Malloc<double>;

  extern template class Malloc<void*>;
  extern template class Malloc<char*>;
  extern template class Malloc<uint16_t*>;
  extern template class Malloc<int16_t*>;
  extern template class Malloc<uint32_t*>;
  extern template class Malloc<int32_t*>;
  extern template class Malloc<uint64_t*>;
  extern template class Malloc<int64_t*>;
  extern template class Malloc<float*>;
  extern template class Malloc<double*>;

}

#endif
```

### Quick search

### Table of Contents

- rang - Colors for your Terminal
- Zstr
- C++ API

«
hide menu

menu
sidebar
»

### Navigation

- index
- toc
- Utils »
- Program Listing for File Malloc.hxx

© Copyright 2021, Enrico Bertolazzi.
Created using Sphinx 4.2.0.
